# Re-organising Junior Doctors During the COVID-19 Outbreak: A Single Centre Experience in the United Kingdom

**DOI:** 10.34172/ijhpm.2020.74

**Published:** 2020-05-12

**Authors:** Anokha Oomman Joseph, Janso Padickakudi Joseph, Jasdeep Gahir, Bernadette Pereira

**Affiliations:** ^1^Department of General Surgery, North Middlesex Hospital, London, UK.; ^2^Department of General Surgery, Broomfield Hospital, Essex, UK.

## Dear Editor,

The coronavirus disease 2019 (COVID-19) outbreak has prompted an unprecedented upheaval in the provision of healthcare services globally. Following timely reporting from Italy,^1^ the United Kingdom also prepared for COVID-19 patients requiring hospital admission and intensive care. As part of this response, multiple issues surrounding staffing have become apparent. In this article, we discuss the experience at the North Middlesex Hospital in London of re-organising junior doctors.

## Central Response

In the UK junior doctors rotate through departments and between hospitals as part of their regional training programmes. These rotations are governed by the General Medical Council (GMC) and separate statutory Statutory Education Bodies (SEBs) in England, Wales, Scotland, and Northern Ireland. In mid-March 2020, the GMC and SEBs announced that the planned rotations for April 2020 would not go ahead, unless local arrangements permitted otherwise.^2^ This would minimise disruption to services by keeping junior doctors in departments in which they were already established members of the healthcare team.

## Re-organisationof Hospital Services and Staff

Plans were made to redeploy members of staff from various specialities to the specialities of Medicine, Intensive Therapy Unit (ITU) and Accident and Emergency (A&E), which were likely to become very busy with the influx of patients. These plans were drawn up rapidly; initial discussion started on March 16,2020 and by March 26, 2020 doctors were deployed on the new rota. At the time of writing, the endpoint of deployment was unknown.

The 16 wards in the hospital, except for ITU, were divided into four zones each containing four wards. Every zone was allocated its own team of roughly 35 doctors; a total of 140 junior doctors were redeployed. Dividing the hospital in this manner enabled staffing decisions to be taken in the most efficient, responsive and flexible manner. There were high staffing levels in anticipation of staff illness or periods of self-isolation. The actual running of the ward would be decided flexibly. Each group of doctors was further sub-divided according to their level of medical experience, which guided the nature of responsibilities they took on the wards. Final year medical students have undergone speed-track graduation and have also commenced working at the end of April 2020. With this multi-pronged re-organisation, North Middlesex Hospital has been able to fully respond to the demands placed on it by COVID-19.

Trainees who are unable to do frontline work due to underlying health conditions (eg, immunosuppression, pregnancy) formed a central team of co-ordinators whose task was to review staffing across the Trust every day and redistribute doctors according to needs.

It was clear from the outset that the surge of COVID-19 patients would spill over from the medical wards to the rest of the hospital. There was a significant reduction in surgical activity – all elective operating and some clinics were cancelled.^3^ It was also noted that since the beginning of the outbreak many junior doctors were either sick or self-isolating. Given the discrepancy in the workload between specialities and the number of sick or isolating doctors, it was quite clear that the junior doctors across the specialities had to be re-organised as part of the COVID-19 response.

All annual leave and study leave was cancelled to ensure that all hands are on deck. Similarly, all appraisals and revalidations were put on hold until October 2020. All specialties apart from medicine, ITU and A&E would run on skeleton staff of consultants and registrars, with no other junior trainees. Specialities such as General Surgery, Urology and Orthopaedics saw patients directly in A&E.

## Supporting Doctors

In order to provide adequate supervision there is a visible consultant presence on every ward. Junior doctors are supported in the clinical environment by the development of clear treatment escalation plans and clear guidelines locally for symptom control. There are also updated local guidelines for critical care and palliative team referrals. The re-organisation prompted an extensive programme of education. New local guidelines addressed clinical protocols, updated communication advice and escalation pathways. Due to the prohibitions on social distancing, this educational programme was delivered wholly online. All other central and local teaching programmes are currently on hold.

Junior doctors are also supported from a mental wellbeing point of view. A space was identified in the hospital which has been converted to a wellbeing room. This is a space open to all staff who need some time out for rest and reflection. A wide range of wellbeing sessions, including mindfulness and workshops on topics like sleep and stress were on offer as part of an effort to maintain staff morale. A clinical psychologist also facilitates peer support groups.

## Conclusion

The COVID-19 outbreak has had a profound impact on the working lives of junior doctors. In our experience as a district general hospital in London, United Kingdom the most significant changes have been: keeping junior doctors in their current post (not allowing for rotations); re-deployment to medicine, ITU and A&E; dividing doctors into ward-based teams; direct access to specialty reviews; new skeleton staff teams for most specialties; cancelling all annual and study leave; supporting junior doctors clinically with direct senior supervision; and supporting junior doctors mental health. Some of these rapid adaptations will be here to stay.

## Ethical issues

Not applicable.

## Competing interests

Authors declare that they have no competing interests.

## Authors’ contributions

All authors contributed equally to this manuscript.

## Authors’ affiliations

^1^Department of General Surgery, North Middlesex Hospital, London, UK. ^2^Department of General Surgery, Broomfield Hospital, Essex, UK.
